# Might culture impact the assessment of handedness in Black participants in neuroscience research?

**DOI:** 10.3389/fnhum.2024.1390881

**Published:** 2024-11-06

**Authors:** Michelle Nwufo, Adaeze Onuoha, Cori Mallory, Joy Stradford, Eran Zaidel, Vickie M. Mays

**Affiliations:** ^1^Los Angeles (UCLA) BRITE Center for Science, Research, and Policy Development, University of California, Los Angeles, Los Angeles, CA, United States; ^2^Los Angeles (UCLA) David Geffen School of Medicine, University of California, Los Angeles, Los Angeles, CA, United States; ^3^Los Angeles (UCLA) School of Nursing, University of California, Los Angeles, Los Angeles, CA, United States; ^4^San Diego State University (SDSU)/UC San Diego Joint Doctoral Program in Clinical Psychology, La Jolla, CA, United States; ^5^Los Angeles (UCLA) Department of Psychology, University of California, Los Angeles, Los Angeles, CA, United States; ^6^Los Angeles (UCLA) Department of Health Policy and Management, University of California, Los Angeles, Los Angeles, CA, United States

**Keywords:** black, culture, diversity, neuroscience, hand dominance, handedness screening assessments

## Introduction

There is growing evidence to suggest cognitive impairment and adverse brain health outcomes are associated with chronic health conditions such as diabetes, hypertension, and cardiovascular disease (Barnes and Bennett, 2014; Lock et al., 2023; National Academies of Sciences et al., 2023). These conditions can contribute to cognitive dysfunction by disrupting physiological feedback mechanisms that regulate oxidative stress (Pugazhenthi et al., 2017), causing fluctuations in systemic blood pressure (Wanleenuwat et al., 2019), or promoting vasoconstriction that impairs cerebral perfusion (Stephan et al., 2017). The higher prevalence of these conditions in the Black population (Ajuwon and Love, 2020; Musemwa and Gadegbeku, 2017; Wang et al., 2021) may be associated with an increased risk of dementias, such as Alzheimer's disease, increasingly seen in Black Americans (Basu and Gujral, 2020; Rajan et al., 2019).

While the compelling scientific need for further evaluation of these connections may be helpful in addressing Black health disparities, neuroscience studies have often been slow to advance such contributions in the United States (U.S.) Black population (Burke et al., 2017; Zuelsdorff et al., 2020). This may stem from underpowered Black participation in neuroscience research (Rutten-Jacobs et al., 2024), which is commonly attributed to historically rooted mistrust in medical research (Otado et al., 2015; Scharff et al., 2010; Webb et al., 2022) or ineffective sampling (Abiodun, 2019; Awidi and Hadidi, 2021). An often-overlooked factor that may further impact the inclusion of Black participants in neuroscience studies is researchers' decision-making processes for participants who report forced hand use on screening assessments.

Handedness screening assessments are a common method for establishing hand dominance in neuroscience studies (Scharoun and Bryden, 2014). Hand “dominance” is characterized by a distinct affinity for the hand that demonstrates the highest proficiency when performing manual tasks (Serrien et al., 2006), while hand “preference” is defined by the hand an individual habitually selects for task performance, independent of proficiency (Chatagny et al., 2013). Handedness is believed to influence an individual's mental and neuropsychological abilities (Johncy et al., 2021). Researchers, particularly in human neuroscience, may selectively analyze the data of right-handed individuals to minimize variance in datasets (Bailey et al., 2019; Willems et al., 2014). Unrealized biases embedded in handedness screening tools may unknowingly facilitate misclassification error for Black participants with a history of culturally influenced handedness practices.

Researchers' understanding of how cultural and religious practices shape handedness in diverse Black communities could affect the internal validity of handedness assessment tools that assess forced hand use. We encountered this issue in a preliminary study on racism and cognitive processing, where assumptions about responses to forced hand use, and its potential link to hand dominance, were susceptible to misunderstandings of the cultural and religious factors influencing forced use. Given the need for greater inclusion in neuroscience research, an examination of the decision-making methods surrounding Black participants' employment of handedness warrants further investigation. Examining how cultural and religious practices are established within the Black population and their relationship to handedness could serve as one intervention to increase researchers' acceptance of Black participants who report a history of forced hand use.

## Understanding cultural/religious right-hand practices of African immigrant populations and their descendants

When assessing handedness in culturally diverse populations, it is useful to establish criteria for including or excluding individuals based on the forced use of the right hand, given the historical preference for right-handedness in formal and functional tasks (De Kovel et al., 2019; Galobardes et al., 1999; Klöppel et al., 2010). In some West African countries like Ghana, Nigeria, and Senegal individuals can display a preference for the right hand when performing tasks that require direct contact with others (Alhassan, 2018). Conversely, in these same regions, the left hand is perceived as dirty or intolerable (Awidi and Hadidi, 2021) and relegated to private tasks such as washing one's body or using the bathroom. In particular, the rare hereditary pattern of left handedness may contribute to its cultural perception as an unlucky trait, leading to public avoidance of left-hand use (Jing, 2020). In other words, the use of the right or left hand for a particular activity may not be a matter of dominance nor preference but an enforced practice honored by one's culture.

The left hand is also subject to stigmatization within the religious teachings of Christianity and Islam, commonly practiced among African and African American communities (Agbiji and Swart, 2015; Park et al., 2020; Simmons, 2008). Christian doctrines have historically associated left handedness with the devil (Hertz, 2013), while Islamic scriptures have regarded the left hand as a symbol of uncleanliness or impurity (Fagard and Dahmen, 2004; Singh and Kundu, 1994). Devoutly religious individuals may therefore refrain from using their left hand during social activities, even for simple gestures like handing over an object or receiving money, as they can have significant social repercussions (Alhassan, 2018). To avoid ostracism, adherents to religious customs must often consciously use the right or left hand in a socially cued manner. Consideration of such practices in the Black diaspora may enhance the efficacy of screening protocols while allowing for the appropriate determination of handedness (Shanunu et al., 2022; Zverev, 2006).

This is important knowledge to incorporate in handedness assessments as a longitudinal analysis of U.S. migration trends revealed that the Black population grew by 20 million over the last four decades (Tamir and Anderson, 2022). Additionally, Tamir (2022) reported that the number of Black immigrants living in the U.S. reached 4.6 million in 2019, a substantial increase from the documented 800,000 in 1980. This growth represents nearly a fifth of the total Black population and it is projected that Black immigrants will contribute to approximately one-third of the overall increase in the U.S. Black population's growth by 2060 (Tamir, 2022). Presently, one in every ten Black Americans is foreign-born and these immigrants often maintain strong religious affiliations that underpin their handedness practices (Mohamed et al., 2021; Shanunu et al., 2022). While such ideologies may originate in Africa, cultural socialization plays a significant role in defining the identity of ethnic-racial populations (Wang et al., 2023). Individuals of African ancestry may retain handedness habits passed down from previous familial generations. With the growing influx of African immigrants to the U.S. (Corra, 2023), an effort to recognize their cultural handedness behaviors is essential to effectively refining research screening tools to ensure the integrity of scientific practices in neuroscience research. Further, it would be worth investigating whether these socialized hand preferences correlate to shifts in brain laterality.

## Integration of cultural competencies in handedness assessments

A primary goal of handedness screening assessments is to determine an individual's hand dominance based on their reported hand preference across a variety of tasks. These assessments draw from a basic inventory to evaluate hand dominance, with some screening tools incorporating additional survey questions that examine the influence of familial factors on handedness (Klöppel et al., 2010). Inventories typically include questions about the direction and degree of hand use for routine activities, such as writing, eating, and throwing, while concurrently assessing how frequently one hand is favored over the other (Oldfield, 1971). While handedness inventories are generally regarded as a reliable instrument for evaluating hand dominance, it is important to heed the caution given by psychologist Richard Charles Oldfield, inventor of the Edinburgh Handedness Inventory (EHI). Oldfield acknowledged that the selection of tasks in such inventories requires greater cultural sensitivity to be universally applicable to participants of differing social groups, including variations in sex, culture, nationality, and socioeconomic status (Oldfield, 1971). For example, researchers translating an English version of the EHI within a Chinese validity study identified tasks in the questionnaire that could be revised to better model Chinese practices. Altering a task description from “knife without a fork” to “knife to cut meat or vegetables,” and replacing “spoon” with “chopsticks” ensured that the language used in the EHI was more aligned with Chinese culture (Yang et al., 2018).

Our team's experimental study on racism and cognitive function among Black males revealed the potential for cultural perspectives to influence handedness assessment responses. Participants born to West African immigrants were more likely to report mixed hand use or forced hand conversion. These individuals were initially excluded from the study by us, resulting in an overall decline in the number of eligible participants. To better understand this occurrence, we asked participants to provide more details about their responses to the questionnaire. We discovered that our handedness assessment may not have accounted for African cultural and religious practices where the right hand is traditionally reserved for formal tasks and the left hand for chores (Alhassan, 2018; Awidi and Hadidi, 2021).

Participants in our study frequently sought clarification on a particular survey question about forced right-hand conversion (see Supplementary material). Our team later identified two distinct interpretations of this question by participants. The first interpretation was perceived as whether a parent ever attempted to convert the participant's true handedness from left to right. Meanwhile, the second interpretation was perceived as whether a parent required the participant to temporarily use a preferred hand during task performance. As a result, responses could have different implications depending on participants' comprehension of the question. For example, marking “yes” to the latter interpretation might indicate that the use of the right hand was enforced short-term for specific tasks rather than as a permanent change in handedness.

On the contrary, marking “yes” may lead researchers to conclude that a participant has spent significant effort in converting their handedness to the right side. This is a critical inference because, if a serious attempt at hand conversion is assumed, scientists may believe that permanent changes to brain laterality have occurred (Siebner et al., 2002). Such an assumption could lead to the potential dismissal of participants from a study. Given this implication, further research is needed to examine how cultural practices influence assessment responses across the broader Black community and whether they impact hand dominance, leading to long-term brain changes.

## Potential culturally sensitive modifications to handedness screening assessments

To improve the internal validity of screening tools, adding questions that address the role of culture and religion could provide a more holistic understanding of handedness in ethnically diverse populations. We suggest incorporating questions designed to determine if a cultural or religious practice might account for a dextral response. We recommend including questions that examine whether:

- Participants experience forced changes in handedness based on cultural or religious beliefs (i.e., were you forced to use your preferred hand for this task due to a cultural or religious belief?).
- Hand preference deviates from hand dominance (i.e., is your preferred hand for this task different from your dominant hand?).
- Frequency of the hand used in tasks is equivalent to the assumption of handedness (i.e., how often do you utilize this task in your daily routine?).

Adopting such changes may enlighten neuroscience researchers about the sociocultural dimensions of handedness. A related concern beyond the screening itself is the unavailability of a scientific workforce that can and will drive a research agenda inclusive of racial/ethnic/religious factors that may matter in neuroscience research. Addressing this requires continued efforts to diversify the workforce and incorporate varied perspectives throughout the research process. One effective approach would be for research teams studying culturally diverse populations to enlist community advisory boards (La Scala et al., 2023). These boards, composed of members representative of the study population, can critically review screening tools, and determine whether tasks and associated survey questions are culturally appropriate.

## Conclusion

To advance our knowledge of how cultural and religious differences impact handedness screening tools, careful assessment of handedness research protocols is needed. In the face of improved tools for determining lateralization, neuroscience researchers should consider including left-handed individuals as stratified samples in cognitive studies, as this may help reveal the impact of handedness on cognitive function. To improve the internal validity of handedness research, future studies should examine the effects of culture and religion on hand dominance and its relationship to structure and function in the brain. Researchers can help mitigate disparities in neuroscience research by carefully evaluating tools for potential bias, and thoroughly assessing study inclusion/exclusion criteria that may unknowingly exclude those who bear a greater burden of health disparities. Neuroscience research stands to benefit from reducing brain health disparities in Black populations by driving research agendas whose criteria and knowledge of diversity is based on inclusive science.
