# Supplementary material for: Might culture impact the assessment of handedness in Black participants in neuroscience research?
Source: Front Hum Neurosci. 2024 Nov 6;18:1390881. doi: 10.3389/fnhum.2024.1390881 (PMC11583062; doi:10.3389/fnhum.2024.1390881)
Supplement: Supplementary file 1 [file Image_1.pdf]

Sex: ______ Subject #: _____

Age: ______

Ethnicity:

_____American Indian or Alaskan Native
_____Asian or Pacific Islander
_____Black, not of Hispanic Origin
_____Hispanic
_____White, not of Hispanic Origin
_____Other____________________

1) Do you consider yourself mostly right-handed, mostly left-handed, or ambidextrous?

2) Which right hand do you INSTINCTIVELY or NATURALLY PREFER to do the following things?

Note: We are not interested in which hand you _can_ use for the following tasks. Rather, we are interested in which hand(s) you would _naturally or instinctively_ _prefer_ to use when both hands are available for your use.

	Left only	Left usually	both	Right usually	Right only
Write					
Draw					
Throw a ball					
Slice bread					
Brush your teeth					
Cut with scissors					
Hold a spoon					
Strike a Match (match)					
Open a box (holding the lid)					
Use a broom					

Oldfield, R.C (1971) The assessment and analysis of handedness: the Edinburgh Inventory. Neuropsychologia, 9 (1):97-113

3) Which hand does your father use predominantly?

4) Which hand does your mother use predominantly?

5) How many of your sisters are right-handed?

6) How many of your sisters are left-handed?

7) How many of your brothers are right-handed?

8) How many of your brothers are left-handed?

9) Did your parents ever try to force you to use your right hand? Yes____ No_____
